# Bronchiectasis and the risk of cardiovascular disease: a population-based study

**DOI:** 10.1136/thoraxjnl-2015-208188

**Published:** 2016-08-29

**Authors:** Vidya Navaratnam, Elizabeth R C Millett, John R Hurst, Sara L Thomas, Liam Smeeth, Richard B Hubbard, Jeremy Brown, Jennifer K Quint

**Affiliations:** 1 Division of Epidemiology and Public Health, University of Nottingham, Nottingham, UK; 2 Faculty of Epidemiology and Population Health, London School of Hygiene and Tropical Medicine, London, UK; 3 Department of Respiratory Medicine, University of Nottingham, Nottingham, UK; 4 Centre for Inflammation and Tissue Repair, University College London, London, UK; 5 Department of Respiratory Epidemiology, Occupational Medicine and Public Health, National Heart and Lung Institute, Imperial College London, London, UK

**Keywords:** Bronchiectasis, Clinical Epidemiology

## Abstract

**Background:**

There are limited data on the burden of cardiovascular comorbidities in people with bronchiectasis. Our cross-sectional study estimates the burden of pre-existing diagnoses of coronary heart disease (CHD) and stroke in people with bronchiectasis compared with the general population. The historical cohort study investigates if individuals with bronchiectasis are at increased risk of incident CHD and stroke events.

**Methods:**

We used primary care electronic records from the Clinical Practice Research Datalink. The cross-sectional study used logistic regression to quantify the association between bronchiectasis and recorded diagnoses of CHD or stroke. Cox regression was used to investigate if people with bronchiectasis experienced increased incident CHD and strokes compared with the general population, adjusting for age, sex, smoking habit and other risk factors for cardiovascular disease.

**Results:**

Pre-existing diagnoses of CHD (OR 1.33, 95% CI 1.25 to 1.41) and stroke (OR 1.92, 95% CI 1.85 to 2.01) were higher in people with bronchiectasis compared with those without bronchiectasis, after adjusting for age, sex, smoking and risk factors for cardiovascular disease. The rate of first CHD and stroke were also higher in people with bronchiectasis (HR for CHD 1.44 (95% CI 1.27 to 1.63) and HR for stroke 1.71 (95% CI 1.54 to 1.90)).

**Conclusion:**

The risk of CHD and stroke are higher among people with bronchiectasis compared with the general population. An increased awareness of these cardiovascular comorbidities in this population is needed to provide a more integrated approach to the care of these patients.

**Key messages**

**What is the key question?**

- Do people with bronchiectasis have more cardiovascular comorbidities compared with the general population and are they at increased risk of incident cardiovascular events?

**What is the bottom line?**

- People with bronchiectasis are at increased risk of cardiovascular disease, which does not appear to be attributable to smoking, other established cardiovascular risk factors or comorbidities associated with the aetiology of bronchiectasis, suggesting that bronchiectasis may be an independent risk factor for cardiovascular disease.

**Why read on?**

- This is the largest population-based study to quantify the association between bronchiectasis and cardiovascular disease, which needs to be highlighted to physicians in order to provide a holistic approach to the care of these patients and health service pathways can be integrated to include management of these comorbidities.

## Introduction

Bronchiectasis is a chronic lung disease characterised by repeated episodes of infection and chronic inflammation.^1^ Current best estimates suggest that almost 20 000 new cases are diagnosed annually, over 300 000 people in the UK have a diagnosis of bronchiectasis at present,^2^ and just under 1000 people die from bronchiectasis each year.^3^

Individuals with other chronic respiratory diseases have been shown to be at increased risk of cardiovascular disease.^4–6^ While these data could be extrapolated to include people with bronchiectasis as they share common risk factors of cardiovascular disease, such as hypoxia and systemic inflammation, there are limited data on the extent to which cardiovascular comorbidities exist in bronchiectasis. Some studies have suggested a high prevalence of cardiovascular disease^7^ and cardiac dysfunction^8^ in people with bronchiectasis and more recently, a case-control study demonstrated that people with bronchiectasis had increased arterial stiffness compared with matched controls.^9^

We used UK primary care data to quantify the burden of cardiovascular comorbidities among people with bronchiectasis compared with the general population. We also determined if individuals with bronchiectasis are at higher risk of first-time cardiovascular events compared with those without bronchiectasis.

## Methods

### Data source

The Clinical Practice Research Datalink (CPRD) is an anonymised primary care database from 625 general practices throughout the UK^10^ (http://www.cprd.com). Information is recorded as part of routine care, from face-to-face consultations and following communication from secondary care. Data used for research need to meet a series of quality checks and have been deemed ‘up to standard’ for research use by CPRD.

Approval had been obtained from the Independent Scientific Advisory Committee, which oversees research involving CPRD data (protocol ref: 13_03R; available on request) and the London School of Hygiene and Tropical Medicine Ethics Committee (LSHTM MSc ethics ref: 012-137).

### Study population

Our study population consisted of all individuals aged over 18 years, alive and contributing to CPRD at any point between 1 January 2004 and 31 December 2011 with at least 1 year of records that were ‘up to standard’ prior to entry into the study.

Previously published pre-specified Read code lists developed by a clinical epidemiologist, who is also a consultant respiratory physician, were used to identify people with bronchiectasis.^2^ Individuals were defined as having bronchiectasis if they were over 18 years, had a diagnosis recorded prior to our index date (a randomly chosen date, which was 25 April 2006) and did not have a diagnosis of cystic fibrosis (CF). We also extracted information on demographic factors, comorbid illnesses associated with the aetiology of bronchiectasis and cardiovascular risk factors (hypertension, diabetes mellitus, hyperlipidaemia, smoking habit, family history of cardiovascular disease). Smoking status was defined using information recorded closest to the index date. Individuals without a record of smoking habit were grouped into a stratum for missing smoking information. Each cardiovascular risk factor was classified as present if the first record for the condition was prior to the index date. Comorbidities associated with the aetiology of bronchiectasis were considered present if the first record of the comorbid illness was either prior to the index date or during the study period.

### Definition of outcomes

Medical records were searched for diagnoses of coronary heart disease (CHD) and/or stroke. The validity of recorded diagnoses has previously been established.^11–13^ CHD was a composite outcome of having at least one recorded diagnosis of angina (including unstable angina), myocardial infarction (MI) or coronary artery bypass graft (CABG). Our definition of stroke included ischaemic or haemorrhagic stroke, transient ischaemic attack and subarachnoid haemorrhage.

### Analysis strategy

We conducted a cross-sectional study to determine if the diagnoses of CHD and stroke were higher in people with bronchiectasis, followed by a cohort study to investigate whether those with bronchiectasis were at increased risk of incident CHD and stroke events. We used the same index date for both analyses, which was 25 April 2006.

### Cross-sectional analysis

The primary outcomes were existing diagnoses of CHD and stroke prior to the index date.

Logistic regression was used to estimate the association between bronchiectasis and CHD and stroke as separate outcomes, adjusting for age, sex and smoking habit as a priori confounders. We repeated the analyses, also adjusting for cardiovascular risk factors to explore to what extent these potentially explained the association between bronchiectasis and cardiovascular disease. Multiplicative interaction terms were applied to investigate effect measure modification by age or sex. The interaction term was retained in the model if provided a better fit for the data, using likelihood ratio testing. These analyses were repeated for the individual CHD outcomes, in turn.

### Historical cohort analysis

First-time diagnoses of CHD and stroke after the index date were considered as incident events in these analyses. We excluded individuals with a previous record of CHD or stroke prior to the index date, as the risk of a second event is likely to differ from the first. All individuals were assigned a start date (which was the index date; 25 April 2006) and a stop date which was the earliest of date of outcome, date of death, date of transfer to another practice or 31 December 2011. Crude rates of CHD and stroke were calculated and Cox regression was used to determine if people with bronchiectasis have an increased rate of first CHD diagnosis or stroke events than those without bronchiectasis. A similar approach was used to assess confounding and effect measure modification, using baseline values for cardiovascular risk factors recorded prior to the index date. Nelson-Aalen methods were used to calculate cumulative incidence of CHD and stroke. The proportional hazards assumption was confirmed using graphical methods.

### Sensitivity analyses

We performed sensitivity analyses to explore the impact of comorbidities associated with the aetiology of bronchiectasis and effect of missing smoking data on the association between bronchiectasis and outcomes. The analyses were repeated excluding individuals with comorbidities associated with the aetiology of bronchiectasis (see online supplementary appendix 1). As our main analyses included individuals without recorded information on smoking habit in a stratum for missing information, the smoking sensitivity analyses were repeated after restricting our dataset to (1) only current smokers, (2) only individuals who never smoked and (3) excluding anyone with missing smoking information. Finally, since CHD and stroke events are fatal and possibly competing risks, we repeated the main historical cohort analyses with a competing risk adjustment.

10.1136/thoraxjnl-2015-208188.supp1 supplementary appendix

Likelihood ratio tests were used for all hypothesis testing. All statistical analyses were conducted using Stata (V.12; Texas, USA).

### Power calculation

The prevalence of CHD and stroke in the UK general population is approximately 3.2% and 1.4%, respectively. With 10 942 people with bronchiectasis and 3.8 million people without bronchiectasis, this study had in excess of 90% power to detect an OR of ≥1.5. For the survival analysis, there are approximately 103 000 MIs and 152 000 strokes each year in the UK. As CPRD covers approximately 8% of the UK population, 6180 MIs and 9120 strokes would be expected to be recorded in CPRD. A total of 66 CHD or stroke events would be required to detect a HR of ≥1.5, with a power of 80% and a significance level of 0.05.

## Results

### Study population

A total of 3 895 710 adults alive and contributing to CPRD on the 25 April 2006 were included in our study population. There were slightly more women (50.8%) and the median age at the index date was 47 years (IQR: 34–62). A total of 10 942 people (0.3%) had a record of bronchiectasis prior to the index date; the majority were female (60.4%) and the median age at diagnosis was 56.5 years (IQR 41.5–67.6). About 33.6% of individuals with bronchiectasis were current smokers (see table 1). About 63% of people with bronchiectasis had one or more comorbidities associated with the aetiology of the disease (see online supplementary appendix 1). The prevalence of risk factors for CHD or stroke was higher in people with bronchiectasis (see table 1).

**Table 1:** Baseline characteristics of people with bronchiectasis and those without bronchiectasis

Characteristic	No. of people without bronchiectasis (%) (n=3 884 770)	No. of people with bronchiectasis (%) (n=10 942)
Sex		
Male	1 910 218 (49.2)	4339 (39.7)
Female	1 974 552 (50.8)	6603 (60.4)
Age category (years)		
19–45	1 777 981 (45.8)	925 (8.5)
45–54	675 485 (17.4)	1143 (10.5)
55–64	613 095 (15.8)	2651 (24.2)
65–74	415 096 (10.7)	3140 (28.7)
≥75	403 113 (10.3)	3083 (28.2)
Smoking status		
Never-smoker	1 640 051 (42.2)	4119 (37.6)
Ex-smoker	649 519 (16.7)	3151 (28.8)
Current smoker	1 397 503 (36.0)	3672 (33.6)
Missing data	197 697 (5.1)	0
Hypertension	691 520 (17.8)	3771 (34.5)
Hyperlipidaemia	250 749 (6.5)	1296 (11.8)
Diabetes	185 964 (4.8)	951 (8.7)
Family history of cardiovascular disease	787 871 (20.3)	2981 (27.2)

### Cross-sectional study

In total, 2417 individuals with bronchiectasis had a diagnosis of CHD or stroke; 12.9% of individuals had a history of CHD and 9.2% had a history of stroke prior to the index date (see table 2). Median time between recording of the CHD event and the index date was 8.3 and 7.7 years in people with and without bronchiectasis, while median time between record of stroke and the index date was 4.1 years in individuals with bronchiectasis and 5.3 years in those without bronchiectasis. After adjusting for age, sex, smoking, hypertension, hyperlipidaemia, diabetes mellitus and family history of cardiovascular disease, the odds of CHD was 33% higher in people with bronchiectasis compared with those without bronchiectasis (OR 1.33, 95% CI 1.25 to 1.41; p<0.001). Recorded diagnosis of CHD in individuals with bronchiectasis varied with age (p<0.001) and was highest in those aged ≤45 years (see table 2). We found no evidence of effect measure modification by gender (p=0.342).

We found an almost twofold increase in recorded diagnoses of stroke in people with bronchiectasis after adjusting for the a priori confounders and cardiovascular risk factors (OR 1.93, 95% CI 1.85 to 2.01; p=0.007) (see table 2). There was no evidence of statistical interaction by age (p=0.171) or sex (p=0.696). All individual CHD outcomes were also more common in people with bronchiectasis compared with the general population (see table 3).

**Table 2:** Adjusted ORs for the association between coronary heart disease or stroke and bronchiectasis

Outcome	No. of people without bronchiectasis (%) (n=3 884 768)	No. of people with bronchiectasis (%) (n=10 942)	Adjusted OR (95% CI)*	Adjusted OR (95% CI)†	Stratum-specific adjusted OR (95% CI)				
					Age category (years)†				
					≤45	46–54	55–64	65–74	≥75
Coronary heart disease	178 944 (4.6)	1409 (12.9)	1.24 (1.16 to 1.31)	1.33 (1.25 to 1.41)	4.43 (2.20 to 8.93)	1.99 (1.44 to 2.76)	1.70 (1.47 to 1.96)	1.35 (1.21 to 1.50)	1.15 (1.05 to 1.26)
Stroke	139 543 (3.6)	1008 (9.2)	1.88 (1.80 to 1.96)	1.92 (1.85 to 2.01)	–	–	–	–	–

*Adjusted for age, gender and smoking.

†Adjusted for age, gender, smoking, diabetes, hypertension, hyperlipidaemia and family history of cardiovascular disease.

**Table 3:** Adjusted ORs for the association between bronchiectasis and individual coronary heart disease outcomes

Outcome	No. of people without bronchiectasis (%) (n=3 884 768)	No. of people with bronchiectasis (%) (n=10 942)	Adjusted OR (95% CI)*	Adjusted OR (95% CI)†	p Value‡
Angina	116 697 (3.0)	936 (8.6)	1.25 (1.16 to 1.34)	1.33 (1.24 to 1.43)	<0.001
Coronary artery bypass graft	8657 (0.2)	45 (0.4)	1.84 (1.63 to 2.13)	1.87 (1.65 to 2.17)	<0.001
Myocardial infarction	78 411 (2.0)	518 (4.7)	1.08 (0.98 to 1.18)	1.11 (1.01 to 1.22)	0.012

*Adjusted for age, gender and smoking.

†Adjusted for age, gender, smoking, diabetes, hypertension, hyperlipidaemia and family history of cardiovascular disease.

‡p Value from likelihood ratio test.

### Historical cohort study

**Figure 1:**
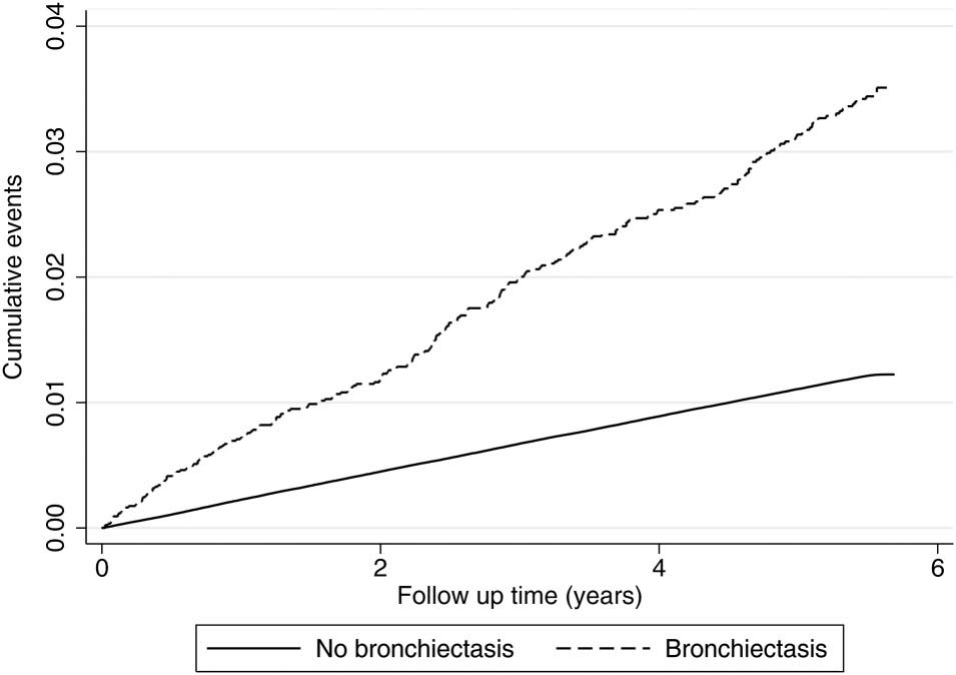
Nelson-Aalen cumulative incidence of coronary heart disease in people with bronchiectasis and those without bronchiectasis is shown.

After excluding individuals with a previous record of CHD or stroke prior to the index date, our cohort consisted of 8622 individuals with bronchiectasis and 3 637 924 individuals without bronchiectasis. The historical cohort was followed up for a median of 5.6 years (IQR: 5.1–5.8), during which there were 40 110 CHD events. The crude rates of first CHD event in people with and without bronchiectasis were 6.6 per 1000 person-years (95% CI 5.9 to 7.5) and 2.2 per 1000 person-years (95% CI 2.1 to 2.3), respectively (see figure 1). The rate of first CHD event was 42% higher in people with bronchiectasis compared with those without bronchiectasis, after controlling for the effects of age, sex and smoking habit (HR 1.42, 95% CI 1.25 to 1.60; p<0.001). Adjusting for diabetes, hypertension, hyperlipidaemia and family history of cardiovascular disease in addition to the a priori confounders had minimal impact on the rate of CHD (HR 1.44, 95% CI 1.27 to 1.63). There was minimal evidence of effect measure modification by age (p=0.181) or sex (p=0.233).

**Figure 2:**
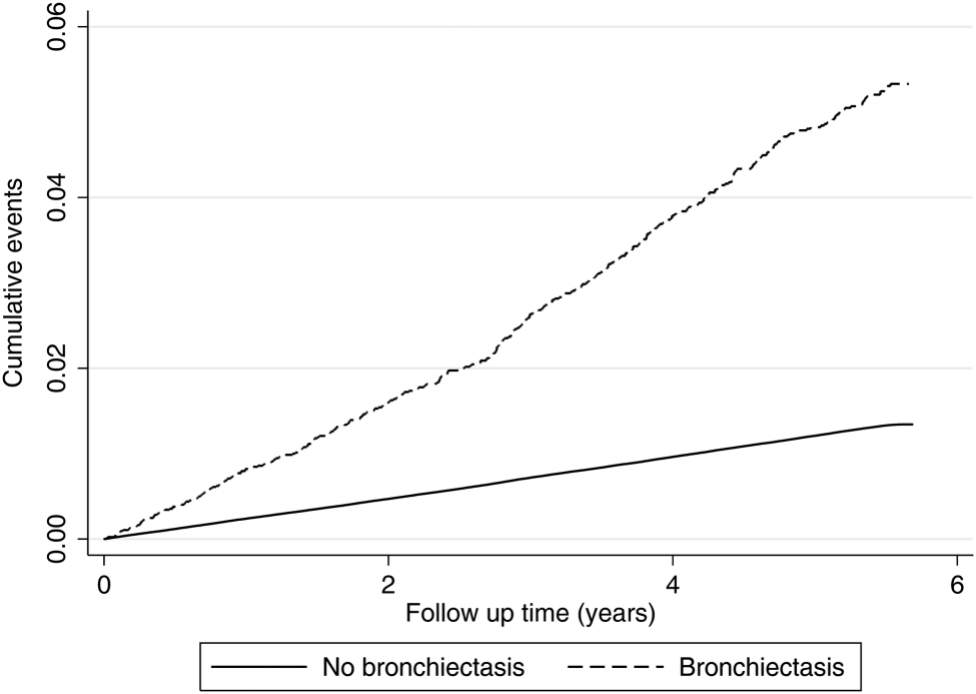
Nelson-Aalen cumulative incidence of stroke in people with bronchiectasis and those without bronchiectasis is shown.

A total of 41 713 strokes occurred during follow-up, which equated to a crude first stroke rate of 9.4 per 1000 person-years (95% CI 8.5 to 10.4) and 2.4 per 1000 person-years (95% CI 2.2 to 2.4) in people with and without bronchiectasis, respectively (see figure 2). After adjusting for age, sex and smoking habit, the rate of first stroke was 69% higher in those with bronchiectasis compared with individuals without bronchiectasis (HR 1.69, 95% CI 1.52 to 1.87; p<0.001). Additional adjustment with cardiovascular risk factors had little effect on the rate of stroke (HR 1.71, 95% CI 1.54 to 1.90; p<0.001). There was limited evidence that the effect of bronchiectasis on acute stroke varied by age (p=0.217) or gender (p=0.105). The data were consistent with the proportional hazards assumption.

### Sensitivity analysis

The sensitivity analyses had marginal change on the ORs and HRs for CHD and stroke (see table 4).

**Table 4:** Adjusted OR and HR for the association between bronchiectasis and coronary heart disease (CHD) or stroke from sensitivity analyses

Sensitivity analysis performed	OR for CHD (95% CI)	OR for stroke (95% CI)	HR for CHD (95% CI)	HR for stroke (95% CI)
Excluding individuals with coexisting diagnosis of COPD (n=7099)*	1.19 (1.09 to 1.29)	1.91 (1.81 to 2.02)	1.36 (1.15 to 1.60)	1.77 (1.55 to 2.02)
Only including individuals without comorbidities associated with the aetiology of bronchiectasis (n=4027)*	1.21 (1.08 to 1.34)	1.93 (1.80 to 2.07)	1.28 (1.08 to 1.48)	1.84 (1.55 to 2.17)
Only including current smokers (n=1 401 175)*	1.29(1.13 to 1.47)	1.79 (1.66 to 1.95)	1.41 (1.10 to 1.79)	1.65 (1.37 to 1.99)
Only including never-smokers (n=1 644 170)*	1.36 (1.23 to 1.50)	1.98 (1.85 to 2.13)	1.47 (1.19 to 1.80)	1.80 (1.51 to 2.14)
Excluding individuals with missing smoking information (n=3 698 015)*	1.35 (1.27 to 1.44)	1.91 (1.84 to 2.00)	1.44 (1.27 to 1.63)	1.71 (1.54 to 1.90)
Competing risk adjustment	n/a	n/a	1.39 (1.22 to 1.59)	1.76 (1.54 to 2.01)

*Adjusted for age, sex, smoking, diabetes mellitus, hypertension, hyperlipidaemia and family history of cardiovascular disease.

## Discussion

This population-based study of almost 4 million people has three main findings. First, we found that individuals with bronchiectasis had higher pre-existing comorbidities of CHD and stroke compared with the general population. Second, there were increased recorded diagnoses of MI and CABG, suggesting that people with bronchiectasis are also likely to have more severe disease requiring intervention. Third, our historical cohort study found that people with bronchiectasis had an increased rate of first CHD and stroke events compared with those without bronchiectasis. Our estimates suggest that one in five individuals with bronchiectasis will have an existing diagnosis of either CHD or stroke and approximately 2500 patients with bronchiectasis will suffer from a first cardiovascular event each year. These individuals are at much higher risk of future vascular events and premature mortality. In absolute terms, our findings suggest that if a cohort of 100 people with bronchiectasis were followed up for 5 years, they would have three CHD events and five strokes, whereas 100 people without bronchiectasis would have one CHD event and one stroke.

There are sparse data on the prevalence of cardiovascular disease or risk factors among people with bronchiectasis. A study of 98 patients with bronchiectasis suggested that patients with bronchiectasis have a high prevalence of cardiovascular-related illnesses: 19.4% had cardiovascular comorbidities, 21.1% had hypertension and 5.1% had diabetes.^7^ A more recent case-control study of 20 cases of bronchiectasis and 20 controls matched for age, sex and smoking status found that cases with bronchiectasis had higher aortic pulse wave velocity, an independent predictor of cardiovascular risk, compared with controls.^9^ We have previously shown that age-specific all-cause mortality rates were higher in people with bronchiectasis compared with the general population, including those under the age of 50.^14^ Although the underlying reason for increased mortality in individuals with bronchiectasis remains unclear, the findings of this study raise the possibility that it could be partly driven by increased cardiovascular disease.

A large proportion of individuals with bronchiectasis in our dataset had comorbidities associated with the aetiology of bronchiectasis, some of which have been associated with increased risk of cardiovascular disease. Our sensitivity analyses only including people with idiopathic or post-infectious bronchiectasis suggest that the increased risk of CHD and stroke is not driven by these comorbidities. We were not able to distinguish between people with idiopathic and post-infectious bronchiectasis, as only a small proportion of our bronchiectasis cohort had specific Read codes for post-infectious bronchiectasis. While it is possible that some of our findings of increased risk of CHD could be due to ascertainment bias, our study also demonstrated increased incidence and prevalence of stroke, which is diagnosed after acute symptoms and confirmation by CT, suggesting that our findings are unlikely to be due to ascertainment bias alone.

The association between CHD and stroke with bronchiectasis may be due a number of reasons; it could be because the diseases share similar risk factors. Studies have suggested that individuals with bronchiectasis have increased systemic inflammation,^15–17^ which is a risk factor for the development of atherosclerosis.^18^ Acute infections which occur more frequently in people with bronchiectasis may also result in a transient increase in the risk of vascular events.^19^ Although possible that the increased risk in CHD and stroke seen in our study is driven by smoking or by comorbidities associated with bronchiectasis, our sensitivity analyses (1) excluding those with comorbid illnesses associated with bronchiectasis and (2) restricted to only never-smokers showed marginal change to our results. While it is also possible that reverse causality may be present, our cohort study only included people without prior cardiovascular events. There is some evidence that chronic multisystem inflammatory disorders are associated with increased risk of cardiovascular disease.^20^ Our findings of an increased risk of CHD and stroke in people with bronchiectasis raises the possibility that systemic inflammation itself is a direct risk factor for vascular disease.

One of the strengths of our study is the large study population, which enabled us to quantify the burden of cardiovascular comorbidities as well as individual CHD events (eg, angina, MI), explore confounding or potential mediation by cardiovascular risk factors and investigate effect measure modification by age and gender. The prevalence of CHD and stroke in our general population is similar to that in national data,^21^ reassuring us of the validity of these diagnoses in our study population, despite the possibility of misclassification. Furthermore, studies have also demonstrated the validity of medical diagnoses^22^ ^23^ and prescribing records in CPRD.^24^ A further strength is the use of prospectively collected data from electronic medical records, which minimises the possibility of misclassification due to recall or observer bias. Although general practices are self-selecting with regard to contributing to CPRD, the population of patients within CPRD is broadly representative of the UK population.^10^

A potential limitation of our study is validity of diagnosis of bronchiectasis. We used a small number of specific previously published Read codes to identify people with bronchiectasis,^2^ only included those aged over 18 years when diagnosed and excluded individuals with a diagnosis of CF to improve the sensitivity and specificity of our bronchiectasis cohort. We were unable to validate the diagnosis of bronchiectasis in our dataset as we did not have access to radiological information. Hence it is not possible to confirm that the diagnoses of bronchiectasis in these data were made according to current guidelines. However, bronchiectasis is usually diagnosed in secondary care after investigation with CT scans^25^ ^26^; hence it seems unlikely for a diagnosis of bronchiectasis to be recorded in primary care without confirmation from secondary care. The prevalence of bronchiectasis in our study population is comparable with other studies and the demographic features are consistent with the UK population of patients with bronchiectasis,^26^ ^27^ providing further reassurance that the diagnosis of bronchiectasis in our dataset is valid. It also needs to be acknowledged that our bronchiectasis cohort is likely to represent a heterogeneous group of patients with a diagnosis of bronchiectasis in their medical records. We did not have information on disease severity or number of hospitalisations for exacerbations and it is possible that the risk of cardiovascular disease may vary with severity of disease. Another potential limitation is missing smoking data in people without bronchiectasis. However, our sensitivity analyses suggest that impact of missing smoking information on the association between bronchiectasis and outcomes is marginal. We assumed that risk factors for CHD and stroke were present if coded and absent if not. However, it is possible that missing data for risk factors were misclassified as absent. Although this may result in some residual confounding, it does not fully explain the association between bronchiectasis and cardiovascular disease. A further possible limitation is that most CHD and stroke events are likely to be diagnosed in secondary care and there may be a delay in recording these events in primary care, particularly at the end of our study period. This may result in an underestimation of the association between bronchiectasis and cardiovascular disease.

Our study suggests that people with bronchiectasis have higher existing cardiovascular comorbidities and a marked increase risk of CHD and strokes. Further research to better understand the biological mechanism behind this association is warranted. With increasing specialisation of secondary care services, clinical awareness of the extent to which cardiovascular comorbidities exist in people with bronchiectasis needs to be raised so that a holistic approach towards patient management can be taken and incorporated into health service models in order to improve patient care.
