# Supplementary material for: Bronchiectasis and the risk of cardiovascular disease: a population-based study
Source: Thorax. 2016 Aug 29;72(2):161–6. doi: 10.1136/thoraxjnl-2015-208188 (PMC5284336; doi:10.1136/thoraxjnl-2015-208188)
Supplement: supplementary appendix [file thoraxjnl-2015-208188supp_appendix.pdf]

Appendix 1

Co-morbid illnesses associated with the aetiology of bronchiectasis

Co-morbid illness		Number (%) (n=10,942)
Allergic Bronchopulmonary Aspergillosis	No	10,782 (98.16)
	Yes	1 (0.01)
	Possible	200 (1.83)
Bone marrow transplant	No	10,936 (99.95)
	Yes	6 (0.05)
Hypogammaglobulinaemia	No	10,831 (99.0)
	Yes	111 (1.0)
Human Immunodeficiency Virus (HIV)	Negative	10,098 (93.3)
	Positive	844 (7.7)
Asthma	No	6288 (57.5)
	Yes	4654 (42.5)
Chronic Obstructive Pulmonary Disease	No	7099 (64.9)
	Yes	3843 (35.1)
Inflammatory Bowel Disease	No	10,648 (97.3)
	Yes	294 (2.7)
Rheumatoid Arthritis	No	10,320 (94.3)
	Yes	622 (5.7)
Other types of connective tissue disease	No	10,375 (94.8)
	Yes	567 (5.2)

*% adds up to more than 100 as some patients had multiple co-morbidities
